# Screen Time and Social Development Through Play in Early Childhood: A Cross-Sectional Study

**DOI:** 10.3390/children13060715

**Published:** 2026-05-22

**Authors:** Maria Cândida de Carvalho Furtado, Waldemar Brandão Neto, Maria Regina Pontes Luz Riccioppo, Isadora Fiacadori Gomes, Paula Saud De Bortoli, Edmara Bazoni Soares Maia, Débora Falleiros Mello

**Affiliations:** 1 Ribeirão Preto College of Nursing, University of São Paulo, Ribeirao Preto 14040-902, Brazil; maria.riccioppo@alumni.usp.br (M.R.P.L.R.); paula.bortoli@usp.br (P.S.D.B.); defmello@eerp.usp.br (D.F.M.); 2 Nossa Senhora das Graças School of Nursing, University of Pernambuco, Recife 52171-011, Brazil; waldemar.neto@upe.br; 3 Hospital das Clinicas, Faculty of Medicine at Ribeirão Preto, University of São Paulo, Ribeirao Preto 14048-900, Brazil; ifgomes@hcrp.usp.br; 4 Paulista School of Nursing, Federal University of São Paulo, Sao Paulo 04024-002, Brazil; ebsmaia@unifesp.br

**Keywords:** child development, mothers, screen time, child care, pediatric nursing

## Abstract

**Highlights:**

**What are the main findings?**

- Peer play was associated with healthier screen time patterns in early childhood.
- Body-based exploratory play and the use of homemade toys were associated with appropriate screen time use.

**What are the implications of the main findings?**

- Encouraging different types of play may help reduce screen time during early childhood.
- Parental involvement in play activities may help reduce screen time in early childhood.

**Abstract:**

Background/Objectives: Excessive screen time has become increasingly common among children worldwide. The current study investigated the relationship between adherence to recommended screen time guidelines and family and child characteristics and social development through play. Methods: This cross-sectional study included 278 mothers from all five Brazilian geographic regions who answered two self-administered online questionnaires assessing sociodemographic characteristics, family characteristics, child development, and screen use in children aged 0–3 years. Analyses included descriptive statistics, chi-square tests, and logistic regression to identify factors associated with adherence to recommended screen time guidelines. Results: Male sex (OR = 3.306, 95% CI: 1.759–6.213), family characteristics (living with both parents, OR = 4.102, 95% CI: 1.134–14.836) and aspects of social development (playing with another child (OR = 2.410, 95% CI: 1.024–5.650); body-based exploratory play (OR = 2.941, 95% CI: 1.225–7.042); and playing with homemade toys (OR = 1.931, 95% CI: 1.032–3.623)) were associated with adherence to recommended screen time guidelines. Conclusions: Appropriate screen time use was associated with male sex, living with both parents, playing with peers, engaging in body-based exploratory play, and using homemade toys. Routine child health consultations must explore family characteristics and evaluate aspects of children’s social development to identify healthy screen use behaviors.

## 1. Introduction

Excessive screen time has become a common behavior among children and adolescents around the world [1]. Indiscriminate access to televisions (streaming platforms), mobile phones, tablets, and computers has led children to change their habits, affecting their quality of life [2]. Evidence regarding children’s screen use points not only to negative repercussions on child development, but also to positive effects on children’s behavior, learning, and socialization [3,4]. In this sense, this study focuses on the use of screens in early childhood and on aspects of child development involving the child’s relationships/interactions with themselves, with family members, and with other children, which may serve as protective against negative developmental outcomes.

Child development (CD) involves continuous permeated changes related to system maturation and skill acquisition [5]. And, as an ongoing process, CD is related to different fields of human behavior (motor, cognitive/language, and psychosocial) [6] that can be interconnected, so that certain skills are learned from restrictions or opportunities for experimentation offered to the child [7]. The first years of a child’s life are extremely important for cognitive, language, and socio-emotional development, and specifically the first three years, which correspond to very early childhood, a period when the child has greater brain plasticity [8]. During this period, neural circuits of the brain are formed and strengthened through stimuli and attachment relationships and lay the foundation for lifelong development [9]. Excessive screen exposure during early childhood affects general cognitive development, particularly by impairing attention to environmental stimuli, social experiences, problem-solving, and communication with others [2].

The guidelines of pediatric societies, including in Brazil [10], in line with the recommendations of the World Health Organization (WHO) [11], suggest that children under 2 years old avoid contact with screens completely, while children aged 2–5 years should be exposed to screens for no more than 1 h per day. However, worldwide, adherence to these recommendations has been low. A meta-analysis indicated that the prevalence of compliance with the screen time guidelines for children under 2 (0 h/day) was 24.7% (1 in 4), and for children 2–5 years old, the prevalence of compliance with the screen time guidelines (1 h/day) was 35.6% (1 in 3) [12]. Studies have demonstrated an association between screen time and negative outcomes in CD, related to child communication and language development [13,14], problem-solving, and socio-personal development that includes the child’s interactions [15]. When screen time exceeds 1 h per day in early childhood, unfavorable results have already been observed for physical, social, and cognitive development [16]. Children who begin excessive screen exposure early are at a greater risk of prolonged use at later ages, developing problematic or addictive pattens of screen use, which entails an additional risk to CD [17].

Recent evidence pointed out that excessive and low-quality screen time [3], without adult supervision, without content guidance, and/or without educational purposes has resulted in lower language skills [18,19,20]. Furthermore, its excessive use reduced emotional and social development [3], with a negative impact on cognitive and social skills, particularly affecting attention, memory, and emotional regulation [21] in very young children.

Although negative repercussions have been demonstrated with the indiscriminate and/or unsupervised use of screens, positive findings highlight that the use of screens supervised by an adult, with a purpose (e.g., educational) and also within the time recommended for each age group has potential to enhance the child’s linguistic, emotional, social and interactional skills, as well as cognitive development [3,4,18,19].

Daily life at home and parenting are essential for monitoring children’s screen time [1]. Although some parents express concern about screen time, there are individual, social, and contextual characteristics related to family structure, communication among family members, and the quality of interactions with the child that influence healthy screen use practices [22]. Interest in the study of screen time in early childhood has grown in recent years, and much of the research has focused on parental caregivers, demonstrating significant evidence based on the influence of parenting [23]. Considering cognitive, social-emotional, and language skills in the early years of life within the context of child development, the research question is: Can interactions with parental caregivers, other children, and independent play be associated with lower screen time among very young children? The present study explores how factors related to CD may affect the appropriate use of screen time, particularly in underrepresented sociocultural contexts. It seeks to increase scientific knowledge about this problem that is present in the lives of children in the modern world. The topic is highly relevant to nurses and other healthcare professionals, as they are involved in child health monitoring in the context of primary health care. These professionals play an important role in detecting vulnerable situations [24] and promoting positive behaviors with parental caregivers [25,26]. The current study aimed to investigate the relationship between screen time practices and family and child characteristics and aspects of social development through play in children aged 0 to 3 years old.

## 2. Materials and Methods

### 2.1. Study Design and Participants

We conducted a cross-sectional study in all 26 Brazilian states and the Federal District, involving mothers as the main caregivers of children aged 0–3 years (very early childhood). A nationwide study was adopted, given Brazil’s large geographic size and the sociocultural diversity across its regions, which may influence knowledge about CD and stimulation opportunities for children. A non-probabilistic sampling strategy was adopted, as well as an online recruitment strategy based on social media platforms (Facebook, Instagram) and a messaging app used by groups of researchers/nurses working with children across different regions of the country. Although this strategy may have excluded participants without digital access, the study aimed to include participants from all the regions of the country. Therefore, online data collection was adopted as the most feasible strategy for nationwide dissemination of the study. The invitation included information about the research objectives, target audience, and the researcher’s contact information. Inclusion criteria were being responsible for the child’s daily care and being over 18 years old. One of the exclusion criteria was the inability to use online data collection tools. This study recruited only children with typical (neurotypical) development; therefore, another exclusion criterion was being the caregiver of a child undergoing specialized follow-up for developmental delay.

### 2.2. Measuring Instruments and Variables

Participants completed two self-administered questionnaires. The first was administered to all of them, with 47 items containing sociodemographic, family, and child characteristics. The questionnaire was developed by one of the authors [M.C.C.F.] and validated by seven researchers with academic and clinical experience and expertise in CD, with an average response time of 25 min. The second questionnaire included statements describing child development milestones reproduced in full from the child’s handbook, which is distributed free of charge (by the Ministry of Health) to all children and used in pediatrics’ healthcare services nationwide [27]. Each statement indicates a milestone. Some examples of the statements in each age group are: 0 to 6 months: ‘If you bring your face 30 cm above the child’s face, the child will clearly look at you’; 7 to 9 months: ‘If you offer your child an object to hold, the child will transfer it from one hand to the other’; 10 to 12 months: ‘If you make a gesture that the child recognizes, such as clapping or waving goodbye, the child will imitate you’; 13 to 15 months: ‘The child walks well, with good balance, without needing support’; 16 to 18 months: ‘The child says three words other than the names of family members or pets’; 19 to 24 months: ‘The child kicks the ball without support’; 25 to 30 months: ‘The child is able to put on some clothing, such as: underwear, socks, shoes, a coat, among others’; 31 to 36 months: ‘The child participates in play with other children of the same age’. Participants selected one of the following responses: “Yes” (the milestone is present), “No” (the milestone is not present), or “I don’t know” if the participant did not identify the milestone. The statements were divided according to the age group indicated on the child’s handbook and the participant responded only to the statements relevant to their child’s age group: 0 to 6 months, 16 statements; 7 to 9 months, 4 statements; 10 to 12 months, 4 statements; 13 to 15 months, 4 statements; 16 to 18 months, 4 statements; 19 to 24 months, 4 statements; 25 to 30 months, 4 statements; and 31 to 36 months, 4 statements.

Additionally, the second questionnaire included two questions for all age groups: “Do you stimulate the child’s development?” with binary response options (yes/no) and “Provide examples of activities used to stimulate the child’s development.” The use of milestones described in the child’s handbook [27] was justified because it is an information source of developmental information; it is routinely used by children and their family, and caregivers are encouraged by healthcare professionals to use it [28,29,30].

The outcome variable (proper screen time) was defined based on WHO guidelines [11] that recommended avoiding screen exposure for children under 2 years of age, and with children 2 to 5 years old, limiting screen time to less than 1 h/day. Given that the sample for this study was composed of children 0 to 3 years old (very early childhood), we followed the recommendation present in the guideline [11] that all children 0 to 2 years old should not use screens and that children between 2 and 3 years old should use them less than 1 h/day. To assess the child’s screen time, the participant selected the option corresponding to the total amount of time the child spent using screen devices, including television, smartphones, or tablets, and/or playing video games during a typical day (my child does not use screens; <1 h per day; 1 to 2 h per day; >2 h per day). Expected developmental milestones were described for each age group, and participants indicated whether each milestone was present in their child.

### 2.3. Data Collection and Ethical Procedures

Interested caregivers contacted the principal investigator via e-mail and chose how they preferred to receive the study materials (Supplementary Material File S1), by receiving a link to the online form (Google Forms) either by email or via a messaging application. All participants chose the text messaging application and provided their phone numbers. After receiving the link to the online form, the participants had access to the informed consent form and the questionnaires described above and had 20 days to complete them.

The participants answered the first questionnaire; at the end of it, there was an item to select the child’s age, and upon doing so, the corresponding page of the second questionnaire containing the age-specific milestones described above became available. The researchers also asked the participants to refer to other eligible caregivers (names and contact information), who were then invited by the principal investigator to participate in the study.

Before each age group and the guidelines on how to answer the questionnaires, the participants were informed that each milestone expected for the child could be present or absent, and the absence of a milestone at a given age would not necessarily indicate a developmental problem. Developmental milestones emerge progressively over time (and are not limited to a specific age range), and if they are absent now, may be achieved later. The child could also present the milestones earlier than expected. Data collection was conducted over a two-month period, considering dissemination on social media, questionnaire distribution, and obtaining the participants’ responses.

The study was conducted in accordance with the Declaration of Helsinki, and the protocol was approved by the Ethics Committee of the University of São Paulo at Ribeirão Preto College of Nursing (5.472.875) on [15 June 2022]. All participants accessed the informed consent form through a secure online link, which contained information about the objectives of the research and data collection. Participants electronically indicated their consent or refusal to participate. The participants later received the informed consent forms signed by the researchers via a text messaging application. At the end of the data collection, the survey link was deactivated. The file with the data was downloaded as a spreadsheet file as previously indicated, and the survey was permanently deleted from the Google Forms platform, to minimize risks to participant privacy and data confidentiality. Additionally, all participant phone numbers were deleted from the principal investigator’s mobile device.

### 2.4. Statistical Analysis

After data collection was completed, the data collected from the questionnaires was exported from Google Forms to a Microsoft Excel spreadsheet. Descriptive statistics were used to describe the distribution of the variables, using absolute and relative frequencies. We used the chi-square test to select variables for inclusion in the logistic regression model to identify bivariate associations between categorical explanatory variables and the binary outcome (appropriate screen time). Outlier analysis was performed, and no influential outliers were identified. All logistic regression assumptions were satisfied. To assess associations between exposure variables and the outcome, crude and adjusted odds ratios (ORs) were estimated using logistic regression. Analyses were performed using SAS software, version 9.4. Statistical significance was defined as *p* < 0.05.

## 3. Results

Between June and August 2022, 296 people received a link to the informed consent form and online questionnaires. Seventeen (5.7%) did not agree to participate after reading the informed consent form, and 279 (94.3%) provided informed consent; however, one participant was excluded because they resided outside the country. Thus, 278 participants were included in the study. Participants were recruited from 23 Brazilian states and the Federal District, with no participants from three states in the North region (Amazonas, Roraima, and Amapá). All participants were the children’s mothers, and the mean maternal age was 29.8 years (SD = 5.305 years); approximately two-thirds had completed university education, and nearly half of the fathers were also reported to have completed university education. Formal employment was reported by more than half of the mothers and about three-quarters of the fathers. Mean household income was USD 1486.32 (minimum USD 78.43; maximum: USD 7843.14).

No mother reported the absence of the developmental milestones expected for her child’s age group.

Based on the study’s focus on early childhood, the 278 children were divided into two groups (≤24 months and ≥25 months), including 142 (51.1%) children aged ≤ 24 months and 136 (48.9%) children between 25 and 36 months.

Table 1 shows that the exposure variables child sex, body-based exploratory play defined as solitary play involving body exploration (e.g., hands, feet, and mouth), using toys made at home, and playing with another child had a statistically significant association and were included in the logistic regression analysis. For the construction of the multivariable model, bivariate analyses were initially performed between the independent variables and the outcome. Variables with a *p*-value < 0.20 were considered eligible for inclusion in the multivariable analysis in order to minimize the premature exclusion of potentially associated factors.

In addition, variables considered theoretically relevant were retained in the model regardless of the statistical significance observed in the bivariate analysis. The definition of theoretical relevance was based on previous evidence from the literature and on conceptual models related to child development, screen time exposure, and social interactions in early childhood, taking into account potential confounding factors and contextual determinants recognized in the field [2,31,32,33].

In the multivariate analysis (Table 2), male children and children engaged in body-based exploratory play were three times more likely to meet recommended screen time guidelines. Additionally, children who played with peers and homemade toys were also more likely to meet the screen time recommendations. After adjusting for the variables, male sex and body-based play remained significantly associated with the outcome. Children under two years old had a 0.4 times lower chance of having screen time within the guideline’s recommendations; children living with both parents had a four times higher chance of appropriate screen use, compared to those who lived with only one parent, in the adjusted model.

## 4. Discussion

Screen use in early childhood is a problem that has been gaining the attention of researchers and healthcare providers, such as nurses and pediatricians, especially after the long period of public health restrictions during the COVID-19 pandemic [5,34]. The proportion of children who use digital media to play, study, and engage in social interactions has been increasing, which requires responsible monitoring and the establishment of limits in the home environment. Excessive screen exposure during early childhood has been associated with lower cognitive abilities and academic performance in later years and reduces opportunities for affectionate interactions in children’s developmental context [35].

In the present study, sociodemographic variables and aspects of social development in very young children demonstrated statistically significant associations with the outcome of appropriate use of screen time. In the sample of mothers in different regions of Brazil, it was found that male sex was associated with higher odds of appropriate screen use, which was consistent with previous studies [36]. In contrast, evidence from Turkey [37] and China [38] demonstrated that greater screen exposure, across digital devices, was more common among boys [37]. This result may be related to cultural differences in parenting styles and practices and to traditional gender roles shaped by sociocultural norms [39]. Research demonstrates that boys spend more time playing outdoors [40] and are more encouraged to engage in physical and leisure activities and to be more sociable, which could contribute to less screen time. On the other hand, findings indicate that girls may be encouraged toward more sedentary and home-based activities, which may increase exposure to screen-based activities [41]. Although caution is needed in asserting a direct relationship when considering gender for screen use, it is important that studies demonstrate trends and even differences in the use of electronic devices between boys and girls. Another Turkish study highlighted the importance of urban green spaces near homes to reduce screen time, especially for girls [42].

Children under 24 months of age had significantly lower odds of adhering to screen time recommendations compared to other children. This disparity can be attributed partly to the different thresholds established by the WHO guidelines of zero-screen policy for children under 2 years versus a one-hour limit for children aged 2 to 3 years. Those lower odds observed in the younger group may reflect the inherent difficulty of achieving screen avoidance in a digitalized domestic environment, as indicated by the compliance trend observed in another study [12].

Family structure plays an important role in screen-based behaviors [43]. In the current study, children who lived with a father and mother were more likely to appropriately use screens than those in single-parent households. A study of Norwegian children and young people found that children living with a single parent or in reconstituted families had a higher risk of excessive screen time, exceeding two hours/day [44]. Previous studies have pointed to benefits for the socio-emotional development of children when parents spend time with them, improving the family climate and increasing affective ties, which favors family functioning [45,46]. This closeness between parents and children can be used to manage children’s screen time. Recent evidence reinforces that screen use under adult supervision, and educational screen use can bring benefits to child development [3,4,18,19].

Children who experience responsive parenting develop better social-emotional skills and autonomy to participate in decisions about acquiring healthier behaviors [24,26]. Studies supported by the “Nurturing Care” model have found that adopting responsive care in early childhood, such as reading, singing, playing, and cuddling, has a positive impact on the child’s holistic development [47], including protection for appropriate screen use [2]. Children under 2 years old are more vulnerable to the negative effects of excessive screen exposure because it causes distraction, disrupts play time, and reduces opportunities for parent–child interaction [48]. Play is the main mode of learning in early childhood [49]. In the current study, children who were encouraged to play with other children used screens appropriately, consistent with other evidence [50], potentially benefiting from peer interactions and social skill development, which contributes to the formation of secure, stable, and stimulating relationships with their caregivers [49]. In a study conducted in the northeast region of Brazil, there was an association between excessive screen exposure and a decrease in the domain related to the child’s ability to play with others, worsening with each additional hour of screen time [15]. Another study demonstrated a negative relationship between children’s interactive and passive screen time and their engagement and participation in active play. Furthermore, it revealed a negative relationship between screen time and social play. The findings provide insights into the connection between children’s engagement and participation in social, interactive, and screen-based educational activities and their engagement and participation in social and active play [51].

The rise in digital play is not inherently harmful; in fact, it offers new possibilities for learning and entertainment. However, when digital play substantially overshadows free play, children may miss out on crucial experiences necessary for their physical health, social competence, and emotional flourishing. Children can become proficient in technology (a valuable modern skill) without sacrificing the creativity, bodily development, and social engagement of traditional play [52]. Parents strongly influence both the quantity and quality of their children’s interactions with other children, for example, adjusting their vocabulary and level of play helps align interactions with the child’s developmental level and ensures a natural environment for the formation of their child’s cognitive and social skills [53].

Excessive reliance on televisions, phones, and other screens to engage young children may limit opportunities for social interaction and active play. One study pointed out that touchscreen tablet games were associated with decreased joint attention among young children; they were less likely to respond to a behavioral request [54]. It is important that parents not only regulate children’s use of digital devices to support healthy child development [2], but also that they offer screen time with interactive and educational content for their children [3,21]. This intentional and educationally oriented offering has a positive influence on the child’s language and executive function development [21] and reinforces the need for balanced consumption of digital media time and content. Prolonged screen exposure may strongly influence children’s attention and self-regulation abilities [55]. Parental behaviors and attitudes, family communications, and social interactions contribute to healthy screen time habits in children [56]. Therefore, nurses must guide parents to limit their children’s and their own screen time and warn them not to adopt severe and restrictive parental practices for digital control [57], because even the environment and digital media play a relevant role in children’s learning [3,21,58]. Digital parenting practices need to strike a balance between the use of technology and other activities by the child, create boundaries and rules around media use, encourage creative and outdoor play and reading, and recognize the autonomy and rights of the child [59].

An important finding of this study is that both body-based exploratory play and play with homemade toys were associated with appropriate screen use. Similarly, an Australian study with children 4–7 years old demonstrated that playing with toys manually and using object substitution in play (e.g., improvised and/or handmade objects) acted as a potentially moderating factor in the impact of screen time [60]. This research suggests that this data warrants further investigation, especially involving the children in early childhood and the environments in which they live (whether favorable or not; with or without resources). The child’s choice of toy has social significance, as it is connected to the child’s cultural context. In other words, toys, objects, and the interest in making them are influenced by the social meaning they assume and engage children in different ways [61]. In another study, the way children played with toys was affected by excessive screen time [15]. Toys that stimulate action, imagination, and learning may support child development more effectively [62]. Children who are actively engaged in activities that require manual functional skills reduce their amount of screen time, and this also contributes positively to sensory processing, visual-motor ability, and fine motor function accuracy [60,63]. The use of homemade toys needs to be further encouraged. A study in a rural area of Zhejiang province, China, found that only 30% of children had access to them (in a sample of 581 children aged 6 to 24 months). In rural or even low-resource environments, play needs to be encouraged by families in contrast to traditional toys [64]. These findings highlight the need to reinforce aspects of psychomotor stimulation that have diminished over time, such as children’s ability to explore their own bodies, while parents have increasingly relied on alternative forms of stimulation and interaction. Playing with one’s own body promotes child awareness and self-discovery, strengthens trust in/attachment to the caregiver, and establishes a pleasant relationship with the world. It is through play and games that involve the use of their own bodies in the exploration of the environment that the child acquires their motor, sensory, cognitive, social, and emotional repertoire [64].

Body-based exploratory play is essential to CD, making it possible for children to interact with the world around them. By processing the information received from the outside world and during play, children acquire body awareness and refine their brain skills, which helps them develop motor and cognitive repertoires essential for their development [65]. Through play, children also promote exploration, creativity, and self-expression. This contributes to their development being shaped based on the quality of the interactions established in social exchanges, supporting future developmental outcomes [66].

In addition to these findings, the present study acknowledges the tendency of parental caregivers to provide socially desirable responses, as well as the systematic overestimation or underestimation of information related to health behaviors. In studies in which parental caregivers participate by answering questionnaires regarding aspects of the child and family context (e.g., family routines, child behaviors, protective caregiving practices, and other sensitive issues), participants may provide responses intended to please the interviewer and gain social approval, as they may fear exposing situations that could result in social or legal repercussions [67]. To minimize this potential bias, participants were encouraged to provide truthful and accurate responses during data collection.

During child follow-up, nurses and pediatricians can use tools to help them assess CD and obtain data that indicate family behaviors that predispose to excessive screen time. In Brazil, these health professionals have access to the child’s handbook as an important tool when caring for children in primary care services [68]. It is considered a child’s health passport, and all Brazilian children receive a child’s handbook at birth, provided free of charge by the Ministry of Health. Because it contains information about children’s health, it must accompany them to any services where they are treated. During childcare consultations, nurses and pediatricians are expected to document the child’s handbook with information about growth and development, nutrition, accident prevention, immunization, among others.

The seventh edition of the child’s handbook [68] addresses the issue of screen time and indicates ways for nurses and pediatricians to talk to caregivers about the impact of screen time on children’s development and interactions with family members. The digital child’s handbook is a recent initiative from the Brazilian Ministry of Health; parents can download the application and track information about their children’s health.

Brazilian nurses and pediatricians have access to this valuable tool that helps them obtain useful data for childcare and allows them to provide evidence-based guidance to caregivers on actions that will promote appropriate use of screens, based on recommendations from national and international organizations.

As practical implications, the current study adds relevant evidence that can contribute to pediatric nursing and medical consultations in the context of primary health care with the aim of integrating efforts to promote safe and effective routine care that is protective of CD. The important role of nurses and pediatricians in the various comprehensive childcare programs in Brazil and around the world is well-known. However, it is extremely relevant to consider family and child characteristics, in addition to the evaluation of aspects of CD, as possible indicators of appropriate screen use. This will offer nurses and pediatricians new guidelines for the identification of the multiple factors that may hinder the achievement of the social, affective, and cognitive skills necessary for the inclusion of children in social participation. Surveillance strategies for CD should include the factors examined in the current study that impact excessive screen exposure in children.

Nurses and pediatricians need to guide parental caregivers in encouraging and actively involving children in games and recreational activities, without requiring substantial financial resources for toys and/or going out of the house. They can manufacture toys and promote games at home to minimize the potential impact of increased screen use time. Finally, nurses and pediatricians can contribute by increasing family awareness about their active role in developing rules to manage children’s screen behavior. Such rules should not be punitive or restrictive but, rather, safely promote the various forms of learning and knowledge construction that children can achieve.

The novelty of this study lies in approaching the topic of screen use in early childhood, linked to CD and the relationships between children and their parental caregivers. And it advances by indicating that nurses and pediatricians can have at their disposal a tool such as the child’s handbook that supports the assessment of children and facilitates guidance for caregivers to offer health guidance during well-child visits.

There are some significant limitations to be highlighted. The data on screen time and developmental characteristics were based on maternal reports, which may be subject to memory bias, considering the possibility of difficulty in accurately recalling the frequency and duration of screen time exposure and the daily routine of children’s play activities. Furthermore, social desirability bias may have influenced participants’ responses, leading to the underreporting of screen time exposure and the overestimation of socially valued practices, such as interactive play activities and family involvement in childcare. To minimize such biases, we used a structured instrument with objective questions and a defined recall period; nevertheless, we acknowledge their potential effects on the magnitude of the observed associations, and the findings should therefore be interpreted with caution. Although the study reached mothers in different regions of Brazil, considering the country’s wide territorial extension, no analysis was performed on the influence of families in vulnerable social, living, and health conditions. Finally, the mothers’ high level of education and probable digital engagement represent other limitations. Therefore, the study’s results cannot be generalized to all parental caregivers in Brazil.

Furthermore, behavioral and social reasons of the families and motivational reasons of the children themselves (i.e., sounds and images from screens are interesting, and the desire to imitate their parents) that attract them to screen use were not explored in this research. Longitudinal studies may provide evidence and consolidate these findings by addressing these questions and identifying factors that may allow for a balance between screen time and optimal cognitive development.

## 5. Conclusions

This study found that the male sex, family characteristics (living with both parents), and social developmental characteristics (peer playing, body-based exploratory play, and play involving homemade toys) were associated with adherence to recommended screen time guidelines in children aged 0–3 years.

In the context of comprehensive pediatric care, nurses and pediatricians must include in their routine child health consultations the combined assessment of screen use and the child development domains examined in this study. Based on these assessments, nurses and pediatricians should identify children who are at greater risk of excessive screen exposure, while recognizing the active role of parental caregivers as key partners in promoting healthy child development.

## Figures and Tables

**Table 1:** Bivariate associations between family and child characteristics and adherence to recommended screen time guidelines.

Variables	Appropriate Time		*p* Value ^a^
	No	Yes	
Sex of the child			
Male	84 (39.62)	42 (63.64)	0.0006 *
Female	128 (60.38)	24 (36.36)	
Ethnicity of the child			
White	150 (70.75)	47 (71.21)	0.9431
Not white	62 (29.25)	19 (28.79)	
Mother’s age (years)			
<29	78 (47.85)	24 (47.06)	0.9211
≥30	85 (52.15)	27 (52.94)	
Mother’s education (years of study)			
<9	73 (34.43)	23 (34.85)	0.9507
≥9	139 (65.57)	43 (65.15)	
Father’s education (years of study)			
<9	113 (54.07)	27 (41.54)	0.0776
≥9	96 (45.93)	38 (58.46)	
Who the child lives with			
Father and mother	184 (86.79)	63 (95.45)	0.0509
Only mother	28 (13.21)	3 (4.55)	
Mother’s occupation			
No employment, homemaker	89 (41.98)	27 (40.91)	0.8774
Formal employment	123 (58.02)	39 (59.09)	
Father’s occupation			
No employment, homemaker	41 (19.52)	15 (23.08)	0.5342
Formal employment	169 (80.48)	50 (76.92)	
Child engages in body-based exploratory play			
No	69 (32.55)	9 (13.64)	0.0028 *
Yes	143 (67.45)	57 (86.36)	
Child plays with homemade toys			
No	81 (38.21)	16 (24.24)	0.0376
Yes	131 (61.79)	50 (75.76)	
Child plays with store-bought toys			
No	7 (3.3)	4 (6.06)	0.3154
Yes	205 (96.7)	62 (93.94)	
Child plays with objects in the house			
No	21 (9.91)	6 (9.09)	0.8452
Yes	191 (90.09)	60 (90.91)	
Caregiver talks to child			
No	85 (40.48)	29 (45.31)	0.492
Yes	125 (59.52)	35 (54.69)	
Caregiver sings to child			
No	60 (28.57)	14 (21.88)	0.2908
Yes	150 (71.43)	50 (78.13)	
Caregiver plays with child			
No	36 (17.14)	10 (15.63)	0.7761
Yes	174 (82.86)	54 (84.38)	
Caregiver reads to child			
No	73 (34.76)	14 (21.88)	0.0525
Yes	137 (65.24)	50 (78.13)	
Child plays with another child			
No	195 (92.86)	54 (84.38)	0.0391 *
Yes	15 (7.14)	10 (15.63)	
Child explores social environment ^b^			
No	147 (70)	41 (64.06)	0.3702
Yes	63 (30)	23 (35.94)	

Note: *: Statistically significant; ^a^: chi-square test with *p* < 0.05, ^b^: Child plays in the yard, on the street, in parks and squares.

**Table 2:** Logistic regression analysis of factors associated with appropriate screen time use according to sociodemographic and child development characteristics.

Sociodemographic Characteristics and Aspects of Child Development	Estimating the Odds Ratio for Appropriate Screen Usage	
	Univariate Analysis	Multivariate Analysis
	OR (95% CI)	AOR (95% CI)
Sex of child (male vs. female)	**2.667 (1.505–4.725)**	**3.306 (1.759–6.213)**
Age of child (<24 months vs. >25 months)	0.618 (0.353–1.082)	**0.416 (0.221–0.784)**
Who child lives with (father and mother vs. only mother)	3.195 (0.939–10.868)	**4.102 (1.134–14.836)**
Child plays with another child (yes vs. no)	**2.410 (1.024–5.650)**	2.146 (0.843–5.464)
Caregiver reads to child (yes vs. no)	0.525 (0.272–1.014)	1.664 (0.816–3.390)
Child engages in body-based exploratory play (yes vs. no)	**3.058 (1.431–6.536)**	**2.941 (1.225–7.042)**
Child plays with homemade toys (yes vs. no)	**1.931 (1.032–3.623)**	1.372 (0.667–2.817)

Note: OR = adjusted odds ratios, fully adjusted for all variables. Bold font indicates that there is a 95% probability that the true odds ratio is likely to fall in the designated range without bias or confusion (statistically significant *p* < 0.05).

## Data Availability

The data presented in this study are available on request from the corresponding author.

## Supplementary Materials

The following supporting information can be downloaded at: https://www.mdpi.com/article/10.3390/children13060715/s1, File S1: Study_Instruments_Child_Dev.pdf: Includes the questionnaires, demographic items, and statements (child development milestones) used in the study.

## Abbreviations

The following abbreviations are used in this manuscript:

CD	Child Development
WHO	World Health Organization
