# Supplementary material for: Screen Time and Social Development Through Play in Early Childhood: A Cross-Sectional Study
Source: Children (Basel). 2026 May 22;13(6):715. doi: 10.3390/children13060715 (PMC13296708; doi:10.3390/children13060715)
Supplement: Supplementary file 1 [file children-13-00715-s001.zip › children-4195985-supplementary.pdf]

STUDY INSTRUMENTS

**Section 1- Child Identification**
Child´s birth date: ______/_____/_______
Sex: [ ] Male [ ] Female
You classified your child's color as:<br>[ ] White<br>[ ] Black<br>[ ] Mixed-race ethnicity<br>[ ] Asian<br>[ ] Indigenous<br>[ ] I don't know
**Section 2- Sociodemographic Data**
**City/State where you live**: _________________
**Maternal age (years old):** _____________
**Mother´s educational level**:<br>[ ] No schooling/illiterate<br>[ ] Incomplete elementary school<br>[ ] Complete elementary school/Incomplete middle school<br>[ ] Complete middle school/Incomplete high school<br>[ ] Complete high school/Incomplete college<br>[ ] Complete college<br>[ ] I don't know/I don´t remember
**Father´s educational level**:<br>[ ] No schooling/illiterate<br>[ ] Incomplete elementary school<br>[ ] Complete elementary school/Incomplete middle school<br>[ ] Complete middle school/Incomplete high school<br>[ ] Complete high school/Incomplete college<br>[ ] Complete college<br>[ ] I don't know/I don´t remember
**Family income (consider all family members) (Value in Reais – R$) __________**
**Marital status:**<br>[ ] single<br>[ ] married/common-law marriage<br>[ ] divorced<br>[ ] widowed
**The child's mother lives:**<br>[ ] with the child's father<br>[ ] with a partner who is not the child's father<br>[ ] without a partner
**Mother´s occupation**:<br>[ ] Homemaker<br>[ ] Formal employment<br>[ ] Informal employment<br>[ ] Unemployed
**Father´s occupation:**<br>[ ] Homemaker<br>[ ] Formal employment<br>[ ] Informal employment<br>[ ] Unemployed

**Section 3 - Child health information**
**The child has an expected development for his/her age:**<br>[ ] no [ ] yes [ ] I don’t know/I don´t remember
**Does the child have the child´s handbook?**<br>[ ] no [ ] yes
**Do you use child´s handbook for:**<br>[ ] I do not use Child handbook<br>[ ] monitor only the child's growth and development<br>[ ] monitor only the child's vaccinations<br>[ ] monitor the child's growth and development + vaccinations
**How often do you take the child´s handbook to the child´s appointments?**<br>[ ] always<br>[ ] sometimes<br>[ ] never<br>[ ] I don’t know/I don´t remember
**How do you seek information about your child's development? (You can choose more than one item):**<br>[ ] I don't seek information, I don't read anything<br>[ ] I talk to my family members<br>[ ] I talk to my friends<br>[ ] I talk to the doctor, the healthcare team that takes care of my child<br>[ ] I check social media (Facebook, Instagram, Twitter, YouTube, WhatsApp)<br>[ ] I read the Child's Handbook<br>[ ] I read books and magazines
**The child plays with (you can choose more than one item):**<br>[ ] their own body (hands, feet, among others)<br>[ ] homemade toys (dolls, cars, balls, among others)<br>[ ] purchased toys<br>[ ] objects from your home (such as pots, pans, plastic objects, among others)<br>[ ] other children<br>[ ] in parks, gardens, outside home
**Does the child use electronic devices such as TVs, computers, cell phones, tablets (listening to music, watching videos and/or programs)?**<br>[ ] no<br>[ ] yes<br>[ ] I don’t know/I don´t remember
**If you answered “yes” (the child uses electronic devices), how many hours a day does the child use them?**<br>[ ] The child does not use these electronic devices<br>[ ] Less than 1 hour per day<br>[ ] 1 to 2 hours per day<br>[ ] More than 2 hours per day<br>[ ] I don’t know/I don´t remember
**How old is the child today? (As you select the age range, you will be directed to another form (this new form has milestones according to the child age)**<br>[ ] 0 to 6 months old (you will be directed to Section 4 – children aged 0 to 6 months old)<br>[ ] 7 to 9 months old (you will be directed to Section 5 – children aged 7 to 9 months old)<br>[ ] 10 to 12 months old (you will be directed to Section 6 – children aged 10 to 12 months old)<br>[ ] 13 to 15 months old (you will be directed to Section 7 – children aged 13 to 15 months old)<br>[ ] 16 to 18 months old (you will be directed to Section 8 – children aged 16 to 18 months old)<br>[ ] 19 to 24 months old (you will be directed to Section 9 – children aged 19 to 24 months old)

Section 4 - STATEMENTS – Child Development Milestones (Child´s Handbook) (Brazil, 2022)

0 to 6 months old

1. The child's arms and legs remain bent, and the head is turned to the side when the child is lying on their back.
[ ] no [ ] yes [ ] I don't know

2. If you bring your face 30 cm above the child´s face, the child will clearly look at you.
[ ] no [ ] yes [ ] I don´t know

3. When you produce a sound (clap your hands or shake a rattle) 30cm from each of the child's ears, without the child being able to see you, the child reacts to the sound stimulus with eye movements and changes in facial expression.
[ ] no [ ] yes [ ] I don´t know

4. The child lifts their head, lifting their chin off the surface, without turning to either side, when the child is lying on their stomach.
[ ] no [ ] yes [ ] I don´t know

5. If you smile or talk to the child (without tickling or touching their face), the child responds with a smile.
[ ] no [ ] yes [ ] I don´t know

6. The child's hands open spontaneously when the child makes certain movements.
[ ] no [ ] yes [ ] I don´t know

7. The child makes some sound (other than crying).
[ ] no [ ] yes [ ] I don´t know

8. The child actively moves their upper limbs (arms) and lower limbs (legs).
[ ] no [ ] yes [ ] I don´t know

9. If you stand in front of the child and talk to them, the child responds with a smile and makes sounds, as if they were talking to you.
[ ] no [ ] yes [ ] I don´t know

10. If you offer an object to the child, touching the back of their hand or their fingers, the child opens their hands and holds the object for a few seconds.
[ ] no [ ] yes [ ] I don´t know

11. If you stand in front of the child and talk to them, the child makes sounds (goo-goo, dada, among others) and laughs loudly, making sounds (giggles).
[ ] no [ ] yes [ ] I don´t know

12. If the child is placed face down on a firm surface, and you attract the child's attention with objects or your face, the child will lift their head and support themselves on their forearms.
[ ] no [ ] yes [ ] I don´t know

13. If you place an object close to the child (on the table or in the palm of their hand) and draw the child's attention to the object, the child will try to reach for it.
[ ] no [ ] yes [ ] I don´t know

14. If you offer an object to the child, the child will put the object in their mouth.
[ ] no [ ] yes [ ] I don´t know

15. If you make a soft noise (bell, rattle, among others) close to the child's ear, the child will turn their head towards the object that produced the sound (on both sides).
[ ] no [ ] yes [ ] I don´t know

16. If the child is placed on a flat surface on their back, the child can roll over onto their stomach (face down).
[ ] no [ ] yes [ ] I don´t know

17. Do you provide stimulation for your child's development?
[ ] no
[ ] yes

18. If you stimulate your child, could you give us some examples (reading books, interacting, talking, singing, playing with the child, among others)? ________________________________

7 to 9 months old

1. If you play peek-a-boo with your child (using your hands, a cloth, or by hiding), the child will make movements to try and find you, such as trying to remove the cloth or turning their face to look for you.
[ ] no [ ] yes [ ] I don´t know

2. If you offer an object to a child to hold, the child will transfer it from one hand to the other.
[ ] no [ ] yes [ ] I don´t know

3. The child says "dad", "mom"
[ ] no [ ] yes [ ] I don´t know

4. If you place a child on a surface and offer them an object to hold, the child will sit without the support of their hands for balance.
[ ] no [ ] yes [ ] I don´t know

5. Do you provide stimulation for your child's development??
[ ] no
[ ] yes

6. If you stimulate your child, could you give us some examples (reading books, interacting, talking, singing, playing with the child, among others)? ________________________________

10 to 12 months old

1. If you make a gesture that the child recognizes, such as clapping or waving goodbye, the child will imitate you.
[ ] no [ ] yes [ ] I don´t know

2. If you place a small object or a crumpled piece of paper near the child and draw the child's attention to pick it up, the child will use a pincer grasp with either part of their thumb or index finger.
[ ] no [ ] yes [ ] I don´t know

3. The child produces incomprehensible conversation alone, with you, or with other people.
[ ] no [ ] yes [ ] I don´t know

4. The child takes a few steps with support.
[ ] no [ ] yes [ ] I don´t know

5. Do you provide stimulation for your child's development??
[ ] no
[ ] yes

6. If you stimulate your child, could you give us some examples (reading books, interacting, talking, singing, playing with the child, among others)? ________________________________

13 to 15 months old

1. The child shows what they want through words or sounds, pointing or reaching out to reach it, without crying.
[ ] no [ ] yes [ ] I don´t know

2. The child puts objects inside a cup or mug.
[ ] no [ ] yes [ ] I don´t know

3. The child says at least one word other than the names of family members or pets.
[ ] no [ ] yes [ ] I don´t know

4. The child walks well, with good balance, without support.
[ ] no [ ] yes [ ] I don´t know

5. Do you provide stimulation for your child's development?
[ ] no
[ ] yes

6. If you stimulate your child, could you give us some examples (reading books, interacting, talking, singing, playing with the child, among others)? ________________________________

16 to 18 months old

1. The child uses a fork or spoon, spilling some of the food outside their mouth.
[ ] no [ ] yes [ ] I don´t know

2. The child places one cube on top of another (stacking) without it falling when the child removes their hand.
[ ] no [ ] yes [ ] I don´t know

3. The child says three words other than the names of family members or pets.
[ ] no [ ] yes [ ] I don´t know

4. The child opens a door or drawer and takes two steps back without falling.
[ ] no [ ] yes [ ] I don´t know

5. Do you provide stimulation for your child's development?
[ ] no
[ ] yes

6. If you stimulate your child, could you give us some examples (reading books, interacting, talking, singing, playing with the child, among others)? ________________________________

19 to 24 months old

1. The child removes an item of clothing, such as: shoes that require effort to remove, coats, pants, or t-shirts.
[ ] no [ ] yes [ ] I don´t know

2. The child stacks three cubes without them falling when the child removes their hand.
[ ] no [ ] yes [ ] I don´t know

3. The child points to two figures in a group of five figures.
[ ] no [ ] yes [ ] I don´t know

4. The child kicks the ball without support.
[ ] no [ ] yes [ ] I don´t know

5. Do you provide stimulation for your child's development?
[ ] no
[ ] yes

6. If you stimulate your child, could you give us some examples (reading books, interacting, talking, singing, playing with the child, among others)? ________________________________

25 to 30 months old

1. The child puts on some clothing, such as: underwear, socks, shoes, a coat, among others.
[ ] no [ ] yes [ ] I don´t know

2. The child stacks six cubes without them falling when the child removes their hand.
[ ] no [ ] yes [ ] I don´t know

3. The child speaks at least two meaningful words in a sentence that indicate an action, such as: "wants water," "wants food," "kicks ball".
[ ] no [ ] yes [ ] I don´t know

4. The child jumps with both feet, landing on the ground simultaneously, not necessarily in the same spot.
[ ] no [ ] yes [ ] I don´t know

5. Do you provide stimulation for your child's development?
[ ] no
[ ] yes

6. If you stimulate your child, could you give us some examples (reading books, interacting, talking, singing, playing with the child, among others)? _________________________________

31 to 36 months old

1. The child participates in play with other children of the same age.
[ ] no [ ] yes [ ] I don´t know

2. The child draws one or more lines on the paper, at least 5 cm long.
[ ] no [ ] yes [ ] I don´t know

3. The child points to pictures according to the action, such as: "who meows?", "who barks?", "who talks?", "who gallops?".
[ ] no [ ] yes [ ] I don´t know

4. The child throws the ball over their arm.
[ ] no [ ] yes [ ] I don´t know

5. Do you provide stimulation for your child's development?
[ ] no
[ ] yes

6. If you stimulate your child, could you give us some examples (reading books, interacting, talking, singing, playing with the child, among others)? ________________________________

PARECER CONSUBSTANCIADO DO CEP

DADOS DO PROJETO DE PESQUISA

Título da Pesquisa: Desenvolvimento da criança e uso de telas na primeiríssima infância: conhecimento e cuidados parentais

Pesquisador: Maria Cândida de Carvalho Furtado

Área Temática:

Versão: 1

CAAE: 58699722.4.0000.5393

Instituição Proponente: Escola de Enfermagem de Ribeirão Preto - USP

Patrocinador Principal: Financiamento Próprio

DADOS DO PARECER

Número do Parecer: 5.472.875

Apresentação do Projeto:

Trata-se da avaliação inicial de um projeto de pesquisa que se caracteriza como exploratório descritivo, do tipo survey.

O estudo buscará por participantes em todo o território nacional. Os potenciais participantes do estudo serão os pais ou cuidadores de crianças com idade de zero a três anos. Tem-se como critérios de inclusão: ser o responsável pelos cuidados e atividades diárias da criança; ter mais de 18 anos. Como critérios de exclusão: não saber ler, o que implica em não conseguir responder os questionários da pesquisa.

Para a obtenção dos dados, serão aplicados dois questionários. O primeiro com dados sociodemográficos, da família e da criança, composto por 47 itens com respostas de múltipla escolha; foi desenvolvido, validado e utilizado em pesquisas anteriores de um dos pesquisadores, com tempo médio de 25 minutos de resposta. Esse questionário é comum a todos os participantes. O segundo questionário contém afirmações sobre os marcos do desenvolvimento infantil da criança. As frases foram extraídas integralmente da Caderneta da Criança. Em cada afirmação, está redigida uma habilidade da criança, e o participante deverá responder “sim” (a criança apresenta a habilidade), “não” (a criança não apresenta a habilidade) ou “não sei”, caso não saiba se a criança apresenta a habilidade. As afirmações foram divididas de acordo com as

habilidades esperadas em cada faixa etária, conforme constam na Caderneta da Criança, e o participante responderá somente as afirmações da faixa etária de sua criança.

Ainda, foram acrescidas em todas as faixas etárias, duas questões finais idênticas; uma delas é se o participante estimula o desenvolvimento da criança, com resposta dicotômica (não ou sim) e outra solicita ao participante redigir exemplos de estímulos realizados com a criança. O tempo de resposta esperado para esse questionário varia de 10 a 15 minutos, considerando o mínimo de 4 e o máximo de 16 afirmações mais os exemplos de estímulos.
Ressalta-se, que antes de cada faixa etária e das orientações sobre como responder os questionários, será indicado que cada habilidade esperada para a criança pode ou não estar presente. E que isso não deve ser motivo de preocupação para o participante quando alguma delas não estiver presente, uma vez que tais habilidades podem se apresentar em um espaço de tempo (não se restringe a uma idade específica), e se não atingida nesse momento, pode se apresentar mais à frente. Como também a criança poderá apresentar a habilidade pouco antes da idade esperada.

Para a identificação dos potenciais participantes (pais/cuidadores) será utilizada a técnica snowball (bola de neve) ou cadeia de informantes. A pesquisa será divulgada em redes sociais (Facebook, Instagram, Twitter), onde constará informações como título da pesquisa, objetivos, critérios de inclusão, como ocorrerá a coleta dos dados e contato com o pesquisador principal. As pessoas que se interessarem em participar entrarão em contato com o pesquisador principal e definirão o modo como desejam ter acesso à pesquisa, com o envio de link ou QRcode do formulário online (Google Forms) por e-mail ou aplicativo de mensagens. Será concedido um prazo de 20 dias para respondê-los, a contar do envio do link ou QRcode.

Objetivo da Pesquisa:

Objetivo Primário:

• Caracterizar famílias de crianças menores de três anos;
• Identificar conhecimento dos cuidadores sobre os marcos do desenvolvimento infantil;
• Descrever estímulos oferecidos às crianças por seus cuidadores.
• Verificar fatores associados ao tempo de uso de telas por crianças menores de três anos;

Avaliação dos Riscos e Benefícios:

De acordo com o documento PB_INFORMAÇÕES_BÁSICAS_DO_PROJETO_1936593.pdf

Riscos: Os riscos de participação no estudo são mínimos e estão relacionados a eventual desconforto dos participantes para responder os questionários. Todos terão liberdade para interromperem sua participação e retornarem quando e se desejarem. Os pesquisadores também asseguram o direito de o participante desistir de participar, sem prejuízos de nenhuma natureza. Conforme indicado no item 3.4 (coleta e registro dos dados), os participantes serão devidamente informados sobre o espaço de tempo (na idade da criança) que se espera que a criança adquira determinada habilidade. Isso para se evitar desconfortos e/ou preocupações, frente à eventual indicação do participante de alguma habilidade não alcançada pela criança. Se desejarem, eles poderão entrar em contato com os pesquisadores para conversarem sobre seu desconforto. Os pesquisadores destacam que uma vez que a coleta de dados se dará exclusivamente por ambiente virtual, há riscos relacionados a este, pelas limitações das tecnologias utilizadas e dos pesquisadores, no que diz respeito à assegurar total confidencialidade e potencial risco de sua violação. Deste modo, os pesquisadores manterão medidas de segurança, sigilo, confidencialidade, armazenamento e proteção de dados. E somente o pesquisador principal e sua orientadora terão acesso ao gerenciamento dos formulários de coleta, com acesso por meio de senha. Ao término da coleta, o pesquisador principal armazenará, em dispositivo pessoal, todas as informações coletadas, sem vínculo com plataformas virtuais. E para segurança e proteção dos dados fornecidos pelos participantes, estas serão apagadas da rede de coleta (Google Forms) para prevenir qualquer extravio de informações.

Benefícios: O benefício direto da participação na pesquisa diz respeito à oportunidade de o participante identificar e também repensar os cuidados oferecidos à sua criança, além de elencar e refletir sobre os principais estímulos que realiza para o desenvolvimento dela e sobre o tempo que a criança usa dispositivos eletrônicos (tempo de uso de tela). Como benefícios indiretos, espera-se que a participação na pesquisa, a sua temática, e a leitura das habilidades esperadas para cada faixa etária (extraídas da Caderneta da Criança) sejam disparadores de desejo de empoderamento dos cuidadores de crianças para se envolverem cada vez mais e buscarem conhecimento constante e estímulos positivos para o DI de suas crianças. Ainda, vê-se como benefício indireto o interesse dos cuidadores em realizarem ou iniciarem o hábito de leitura da Caderneta da Criança.

Comentários e Considerações sobre a Pesquisa:

Vide o campo Conclusões ou Pendências e Lista de Inadequações

Considerações sobre os Termos de apresentação obrigatória:

Foram apresentados:

- folha de rosto devidamente preenchida e assinada;
- cronograma com duração de 9 meses;
- projeto de pesquisa detalhado;
- orçamento no valor de R$ 170,00;
- TCLE para os cuidadores, apresentando todos os esclarecimentos e garantias necessários;
- Aparência do TCLE e formulário de coleta de dados no Google Forms;

Recomendações:

Vide o campo Conclusões ou Pendências e Lista de Inadequações

Conclusões ou Pendências e Lista de Inadequações:

O CEP-EERP/USP considera que o protocolo de pesquisa ora apresentado contempla os quesitos éticos necessários, estando apto a ser iniciado a partir da presente data de emissão deste parecer.

Em atendimento ao subitem II.19 da Resolução CNS 466/2012, cabe ao pesquisador responsável pelo presente estudo elaborar e apresentar relatórios parcial e final "[...] após o encerramento da pesquisa, totalizando seus resultados", em forma de "notificação". O modelo de relatório do CEP-EERP/USP se encontra disponível em:

http://www.eerp.usp.br/research-comite-etica-pesquisa-relatorio/

Considerações Finais a critério do CEP:

Parecer apreciado na 300ª Reunião Ordinária do CEP-EERP/USP.

Este parecer foi elaborado baseado nos documentos abaixo relacionados:

Tipo Documento	Arquivo	Postagem	Autor	Situação
Informações Básicas do Projeto	PB_INFORMAÇÕES_BÁSICAS_DO_PROJETO_1936593.pdf	12/05/2022 11:46:17		Aceito
Orçamento	Orcamentocorreto.pdf	12/05/2022 11:45:59	Maria Cândida de Carvalho Furtado	Aceito
Cronograma	Cronogramacorreto.pdf	12/05/2022 11:45:46	Maria Cândida de Carvalho Furtado	Aceito
Outros	FormularioGoogleForms.pdf	01/05/2022 15:02:09	Maria Cândida de Carvalho Furtado	Aceito

TCLE / Termos de Assentimento / Justificativa de Ausência	TCLECuidadores.pdf	01/05/2022 15:01:40	Maria Cândida de Carvalho Furtado	Aceito
Outros	OficioCEP.pdf	01/05/2022 15:01:08	Maria Cândida de Carvalho Furtado	Aceito
Projeto Detalhado / Brochura Investigador	Desenvolvimentocriancausotelasprimeirissimainfancia.pdf	01/05/2022 15:00:40	Maria Cândida de Carvalho Furtado	Aceito
Folha de Rosto	FRDesenvolvimentocriancasusotelas.pdf	01/05/2022 15:00:16	Maria Cândida de Carvalho Furtado	Aceito

Situação do Parecer:
Aprovado

Necessita Apreciação da CONEP:
Não

RIBEIRAO PRETO, 15 de Junho de 2022

Assinado por:
RONILDO ALVES DOS SANTOS
(Coordenador(a))

Termo de Consentimento Livre e Esclarecido
Cuidadores de Crianças menores de 3 anos

Prezado(a) Senhor(a)

O meu nome é (*nome da pesquisadora*), sou aluna de enfermagem da (*nome da Universidade à qual estão vinculadas as pesquisadoras*). Eu desenvolvo a pesquisa intitulada "**Desenvolvimento da criança e uso de telas na primeiríssima infância: conhecimento e cuidados parentais**", sob orientação da enfermeira (*nome da pesquisadora - supervisora*).

Gostaria de convidá-lo(a) a participar dessa pesquisa, que tem os objetivos: 1) caracterizar famílias de crianças menores de três anos; 2) identificar conhecimento dos cuidadores sobre os marcos do desenvolvimento infantil; 3) descrever estímulos oferecidos às crianças; 4) verificar fatores associados ao tempo de uso de telas por crianças menores de três anos.

Os "marcos do desenvolvimento infantil" significam as habilidades em cada idade (capacidade que a criança tem para realizar uma determinada tarefa ou ação, como sentar, pegar um objeto com as mãos, emitir sons). E o termo "tela" significa dispositivos (aparelhos) eletrônicos como televisão, computadores, tablets, celulares.

O **benefício direto** da sua participação na pesquisa diz respeito à oportunidade de você identificar e também repensar os cuidados oferecidos à sua criança, além de elencar (listar) e refletir sobre os principais estímulos que realiza para o desenvolvimento dela e sobre o tempo que a criança usa dispositivos (aparelhos) eletrônicos (tempo de uso de tela). Como **benefícios indiretos**, espera-se que a sua participação na pesquisa e a temática do desenvolvimento infantil, como também a leitura das habilidades esperadas para cada faixa etária que estão no questionário da pesquisa possam despertar em você o desejo de conhecer mais sobre o desenvolvimento infantil e sobre os estímulos positivos para esse desenvolvimento na sua criança. Ainda, esperamos despertar o seu interesse em ler com frequência a Caderneta da Criança, um importante documento sobre a saúde da sua criança (com dados de crescimento e desenvolvimento da sua criança).

A sua participação será para responder 2 questionários, um deles sobre dados da família e da criança, com 47 questões e tempo médio de 25 minutos para responder. O segundo questionário tem de 4 a 16 frases afirmativas sobre o desenvolvimento de sua criança, com tempo médio de 10 a 15 minutos para responder. Assim, estimamos que você leve de 35 a 40 minutos para contribuir com a pesquisa; esse tempo pode variar para mais ou para menos. Você receberá um link com os questionários e vamos te dar 20 dias para responder a pesquisa; se você precisar de mais tempo, por favor nos avise por correio eletrônico (*endereço de e-mail das pesquisadoras*).

A sua participação envolve riscos mínimos e estão relacionados a eventual desconforto que você pode sentir para responder os questionários. Você tem liberdade para interromper sua participação e retornar a participar da pesquisa quando e se desejar. Você tem o direito de desistir de participar, sem prejuízos de nenhuma natureza. E se desejar, você pode entrar em contato com os pesquisadores para conversar sobre seu desconforto, nos contatos indicados no final desse Termo.

Você também pode não responder qualquer questão (mesmo que sinalizada como obrigatória) e retirar o seu consentimento, sem a necessidade de explicação ou justificativa. Também destacamos que você não terá custos em dinheiro ou receberá algum valor em dinheiro para participar da pesquisa. Entretanto, fica assegurado seu direito à indenização conforme as leis vigentes no país, caso ocorra dano decorrente de sua participação, por parte do pesquisador e das instituições envolvidas nas diferentes fases da pesquisa. Os resultados desse estudo serão publicados por meio de artigos científicos, eventos científicos, mídia e similares. E você também pode ter acesso às suas respostas e ao resultado do estudo; se desejar, enviamos por correio eletrônico.

Ressaltamos que existem potenciais riscos relacionados ao ambiente virtual de coleta de dados, em função de limitações das tecnologias utilizadas e limitações dos pesquisadores para assegurar total confidencialidade e potencial risco de sua violação. Para isso, nos comprometemos a manter todas as medidas de segurança, segredo, armazenamento e proteção de dados possíveis para que apenas eu e minha orientadora tenhamos acesso as informações disponibilizadas por

você. Assim que finalizarmos a coleta de dados, todas as informações serão armazenadas em dispositivo pessoal do pesquisador principal, sem vínculo com plataformas virtuais e as informações serão apagadas das redes de coleta para prevenir qualquer extravio de informações e caso exista alguma violação destes dados, você será informado imediatamente.

Destacamos que ao aceitar participar da pesquisa, nós disponibilizaremos a você um link que contém os questionários da pesquisa e um arquivo referente a este Termo de Consentimento Livre e Esclarecido devidamente assinado pelos pesquisadores responsáveis. É muito importante que você guarde em seus arquivos pessoais uma cópia deste documento eletrônico.

Essa pesquisa foi aprovada pelo Comitê de Ética em Pesquisa da (*nome da instituição à qual estão vinculadas as pesquisadoras*), que tem a finalidade de defender os interesses dos participantes da pesquisa e proteger os participantes em sua integridade e dignidade. Se desejar, você pode entrar em contato com este Comitê, que funciona em dias úteis, das 10h às 12h e das 14h às 16h, de segunda a sexta-feira, na (*endereço e telefone do Comitê de Ética em Pesquisa*).

Para se comunicar com o pesquisador ou sua orientadora, sobre qualquer dúvida ou esclarecimento, entre em contato com (*nome da pesquisadora*) ou com a Enfermeira (*nome da pesquisadora – supervisora*) através dos e-mails, telefones ou endereço abaixo indicados.

Agradecemos a sua colaboração e colocamo-nos à disposição para qualquer informação.

( ) Eu li, concordo em participar da pesquisa e desejo receber, por e-mail, uma via deste termo assinada pelos pesquisadores.

( ) Eu li e não tenho interesse em participar da pesquisa

Nome da pesquisadora
Aluna de Enfermagem
Pesquisador principal
e-mail: *endereço de e-mail*

Nome da pesquisadora - supervisora
Enfermeira. (*Número do Registro de Enfermeira*
Orientadora
e-mail: *endereço de e-mail*
telefone: *número telefone*

Endereço da instituição à qual as pesquisadoras estão vinculadas
(avenida, número, prédio e número da sala da supervisora, CEP, telefone)

Informed Consent Form (translated into English)
Caregivers of Children Under 3 Years Old

Dear Sir/Madam,

My name is (*researcher´s name*), and I am a nursing student at University (*Name of the university to which the researchers are affiliated*). I am conducting research entitled "Child Development and Screen Use in Early Childhood: Knowledge and Parental Care," under the guidance of nurse (*researcher´s name - supervisor*).

I would like to invite you to participate in this research, which has the following objectives: 1) to characterize families of children under three years old; 2) to identify caregivers' knowledge about child development milestones; 3) to describe stimuli offered to children; 4) to verify factors associated with screen time for children under three years old.

The "child development milestones" refer to the skills at each age (the child's ability to perform a given task or action, such as sitting, picking up an object with their hands, making sounds). And the term "screen" means electronic devices such as televisions, computers, tablets, and cell phones.

The direct benefit of your participation in the research is the opportunity for you to identify and rethink the care you provide to your child, as well as to list and reflect on the main stimuli you provide for their development and the amount of time your child spends using electronic devices (screen time). As indirect benefits, we expect that your participation in the research and the theme of child development, as well as reading the expected skills for each age group in the research questionnaire, will awaken in you the desire to learn more about child development and the positive stimuli for this development in your child. We also hope to spark your interest in frequently reading the Child's Booklet, an important document about your child's health (with data on your child's growth and development).

Your participation will involve answering 2 questionnaires, one about family and child data, with 47 questions and an average time of 25 minutes to answer. The second questionnaire has 4 to 16 affirmative statements about your child's development, with an average time of 10 to 15 minutes to answer. Thus, we estimate that it will take you 35 to 40 minutes to contribute to the research; this time may vary. You will receive a link to the questionnaires, and we will give you 20 days to answer the survey; if you need more time, please let us know by email (*researchers´ e-mail address*).

Your participation involves minimal risks, and they are related to any discomfort you may feel when answering the questionnaires. You are free to interrupt your participation and return to participate in the research whenever and if you wish. You have the right to withdraw from participation without any prejudice. If you wish, you can contact the researchers to discuss your discomfort using the contact information provided at the end of this document.

You can also choose not to answer any question (even those marked as mandatory) and withdraw your consent without needing to provide an explanation or justification. We also emphasize that you will not incur any monetary costs or receive any monetary compensation for participating in the research. However, you are guaranteed the right to compensation under the laws of our country should any harm result from your participation, on the part of the researcher and the institutions involved in the different phases of the research. The results of this study will be published through scientific articles, scientific events, media, and similar means. You can also access your answers and the study results; if you wish, we can send them to you by email.

We emphasize that there are potential risks related to the virtual data collection environment, due to limitations of the technologies used and limitations of the researchers in ensuring total confidentiality and the potential risk of its violation. Therefore, we are committed to maintaining all possible security, confidentiality, storage, and data protection measures so that only I and my supervisor have access to the information you provide. Once we finish collecting the data, all information will be stored on the principal investigator's personal device, without any link to virtual platforms, and the information will be deleted from the collection networks to prevent any loss of information. In the event of any data breach, you will be informed immediately.

We would like to emphasize that by agreeing to participate in the research, we will provide you with a link containing the research questionnaires and a file relating to this Informed Consent Form duly signed by the responsible researchers. It is very important that you keep a copy of this electronic document in your personal files.

The Research Ethics Committee of (*Name of the university to which the researchers are affiliated*) approved this research. This Committee aims to defend the interests of research participants and protect their integrity and dignity. If you wish, you can contact this Committee, which operates on weekdays, from 10 am to 12 pm and from 2 pm to 4 pm, Monday to Friday, at (*Address, phone number, and email of the Ethics Committee*).

If you wish to communicate with the researcher or their supervisor regarding any questions or clarifications, please contact (*researcher´s name*) or (*researcher´s name – supervisor*) via the emails, phone numbers, or address indicated below.

We appreciate your collaboration and we are available for any further information.

( ) I have read and agree to participate in the research and wish to receive, by email, a copy of this agreement signed by the researchers.

( ) I have read and am not interested in participating in the research

____
Researcher´s name
Nursing student
Main researcher
e-mail: *e-mail address*

____
Researcher´s name - supervisor
Nurse. *Nurse´s registration number*
Supervisor
e-mail: *e-mail address*
telefone: *phone number*

Address of the University to which the supervisor affiliated
(avenue, number, building and supervisor´s office number, zip code, telephone)
